# Improvement of dyspnea after bariatric surgery is associated with increased Expiratory Reserve Volume: A prospective follow-up study of 45 patients

**DOI:** 10.1371/journal.pone.0185058

**Published:** 2017-09-20

**Authors:** Louis Boissière, Jeanne-Marie Perotin-Collard, Eric Bertin, Isabelle Gaubil, Ana Diaz Cives, Coralie Barbe, Sandra Dury, Julie Nardi, François Lebargy, Gaëtan Deslée, Claire Launois

**Affiliations:** 1 Department of Respiratory Diseases, Reims University Hospital, Reims, France; 2 INSERM UMRS 903, Reims University Hospital, Reims, France; 3 Department of Nutrition, Reims University Hospital, Reims, France; 4 Department of Gastroenterological Surgery, Reims University Hospital, Reims, France; 5 Clinical Research Unit, Reims University Hospital, Reims, France; 6 EA 4683, University of Reims, Reims, France; Medical University of Vienna, AUSTRIA

## Abstract

**Objectives:**

To assess the effects of bariatric surgery in patients with obesity on dyspnea and to analyze the relationships between improvement of dyspnea after bariatric surgery and changes in pulmonary function, especially Expiratory Reserve Volume (ERV) which is the lung volume abnormality most frequently associated with obesity.

**Methods:**

Forty-five patients (5 males/40 females, mean Body Mass Index = 46.2 ± 6.8 kg/m^2^) were evaluated before and 6 to 12 months after bariatric surgery. Dyspnea was assessed by the modified Medical Research Council (mMRC) scale. Pulmonary function tests, arterial blood gases and six-minute walk test were performed. Laboratory parameters including C-Reactive Protein (CRP) were analyzed.

**Results:**

Ninety percent of patients were dyspneic before surgery (mMRC scale ≥ 1) versus 59% after surgery (p<0.001). Mean mMRC score improved after bariatric surgery (1.5 ± 0.9 vs 0.7 ± 0.7, p<0.0001). Among patients with dyspnea before surgery (n = 38), a more marked increase in ERV after surgery was observed in patients with improvement of dyspnea compared to patients with no improvement of dyspnea (+0.17 ± 0.32 L vs +0.49 ± 0.35 L, p = 0.01). Multivariate analysis including age, variation of BMI, variation of CRP, variation of Total Lung Capacity and variation of ERV demonstraded that ERV was the only variable associated with improvement of the mMRc score after bariatric surgery (p = 0.04).

**Conclusion:**

Weight loss associated with bariatric surgery improves dyspnea in daily living. This improvement could be partly related to increased ERV.

## Introduction

Obesity, defined as a Body Mass Index (BMI) greater than or equal to 30 kg/m^2^, represents a significant public health issue. The number of overweight and obese adults (BMI ≥ 25 kg/m^2^) in the world has more than doubled since 1980 and increased from 921 million in 1980 to 2.1 billion in 2013 [1]. In 2013, one-third of the adult population in North America and one-quarter of the adult population in Western Europe suffered from obesity [2].

Effects of obesity on lung volumes are well known and the abnormality most frequently associated with obesity is decreased Expiratory Reserve Volume (ERV), which is exponentially correlated with increased BMI [3], while Total Lung Capacity (TLC) is usually normal [4,5].

Dyspnea is a very common and crippling symptom in obesity. About 80% of people with obesity experience dyspnea in daily living [6,7]. Collet *et al* showed that dyspnea assessed by BDI (Baseline Dyspnea Index) scale was higher in individuals with BMI ≥ 49 kg/m^2^ than in individuals with BMI < 49 kg/m^2^ [8]. Individuals with obesity who experience breathlessness during activities of daily living according to the mMRC (modified Medical Research Council) scale also have a higher BMI, cover a shorter distance on the six-minute walk test (6MWT) and, interestingly, have a lower ERV than individuals with obesity not associated with dyspnea [7]. This observation suggests that the decrease in ERV in obesity, mainly due to the effect of abdominal contents on diaphragm position, could be involved in the dyspnea experienced by individuals with obesity.

Bariatric surgery has been demonstrated to be an excellent treatment for obesity by inducing significant weight loss, ranging from 29% six years after Roux-and-Y gastric bypass [9] to 38% three years after sleeve gastrectomy [10]. There is strong evidence supporting an increase in ERV after weight loss [11]. Studies have also shown that dyspnea at the end of a 6MWT assessed by Borg scale improves after bariatric surgery and that the distance covered on the 6MWT increases by up to 20% [12,13]. Nevertheless, to our knowledge, changes in dyspnea in daily living after bariatric surgery and the links between variations in dyspnea and lung function tests after bariatric surgery have not been previously investigated.

The primary objective of this study was to determine if bariatric surgery is associated with an improvement of dyspnea in daily living according to the mMRC scale in patients with obesity. The secondary objectives were to assess the effects of bariatric surgery on lung function tests (spirometry, plethysmography, arterial blood gases and 6MWT) and the relationships between improvement of dyspnea and changes in lung function tests (especially ERV) after bariatric surgery. We also investigated the effects of bariatric surgery on various laboratory parameters and the relationships between dyspnea and changes in laboratory parameters after surgery.

## Methods

### Patients and design

Adult patients with obesity from the Reims University Hospital Department of Nutrition (France) were systematically evaluated for respiratory symptoms and lung function before bariatric surgery. Indications for bariatric surgery complied with the following French National Authority for Health (HAS) guidelines [14]: age ≥ 18 years and morbid obesity defined as a BMI ≥ 40 kg/m^2^ or BMI between 35 and 40 kg/m^2^ associated with one or more comorbidities such as diabetes, hypertension, obstructive sleep apnea syndrome or other chronic respiratory disease, nonalcoholic steatohepatitis or bone and joint disease. Patients consecutively referred to our department between January 2014 and October 2015 for systematic respiratory evaluation before bariatric surgery and who underwent bariatric surgery were considered for inclusion in this study. Patients were excluded if they did not attend the postoperative evaluation or if they presented with lung or neuromuscular disease. Patients were informed in writing and gave their consent to participate. The study was approved by the Reims University Hospital Institutional Review Board (IRB-1710-2012).

Lung function tests were performed before and 6 to 12 months after bariatric surgery.

### Clinical characteristics and dyspnea assessment

Demographic data (age, sex), BMI, waist and hip measurements, comorbidities, treatments, smoking status and type of bariatric surgery (sleeve gastrectomy or gastric bypass) were systematically recorded. Dyspnea in daily living was evaluated using the mMRC scale (Table 1). The mMRC scale consists of five statements that almost entirely describe the range of dyspnea from none (grade 0) to almost complete incapacity (grade 4). The mMRC scale is the most commonly used validated scale to assess dyspnea in daily living in chronic respiratory diseases. A systematic review of measures used for the sensation of breathlessness in obesity had shown that the mMRC scale can be recommended in obese people based on its reliability and concurrent validity [15].

**Table 1:** Modified Medical Research Council (mMRC) dyspnea scale.

Grade of dyspnea	Description
0	I only get breathless with strenuous exercise
1	I get short of breath when hurrying on level ground or walking up a slight hill
2	On level ground, I walk slower than people of the same age because of breathlessness, or I have to stop for breath when walking at my own pace on the level
3	I stop for breath after walking about 100 yards or after a few minutes on level ground
4	I am too breathless to leave the house or I am breathless when dressing

### Lung function tests

Pulmonary function tests (PFTs) were performed according to the American Thoracic Society/European Thoracic Society guidelines [16,17] (BodyBox 5500 Medisoft Sorinnes, Belgium). Forced Expiratory Volume in one second (FEV_1_), Vital Capacity (VC), Forced Vital Capacity (FVC), FEV_1_ /VC, Inspiratory Capacity (IC) and Expiratory Reserve Volume (ERV) were measured during spirometry. Residual Volume (RV), Functional Residual Capacity (FRC) and Total Lung Capacity (TLC) were measured during plethysmography. As the plethysmography cabin cannot be used in patients weighing more than 150 kg, only spirometry, including FEV_1_, VC, FVC, FEV_1_ /VC, IC, and ERV, were performed in patients weighing more than 150 kg. Results are expressed in Liters and as percentage of predicted values. Carbon monoxide diffusing capacity of the lung (DLCO) was also measured [18].

Arterial blood gases were measured in the morning in the sitting position. The 6MWT was performed according to the American Thoracic Society guidelines (ATS 2002) [19]. Patients were instructed that the objective was to walk as far as possible in 6 minutes. The 6MWT was performed in a 30-meter long, flat, covered corridor, marked meter-by-meter. Heart rate, oxygen saturation and modified Borg scale subjectively assessing the degree of dyspnea graded from 0 to 10, were collected at the beginning and at the end of the 6MWT. The distance covered was calculated at the end of the test.

### Laboratory parameters

After fasting for 12 hours, Aspartate Aminotransferase (ASAT), Alanine Aminotransferase (ALAT), Gamma-Glutamyltransferase (GammaGT), Alkaline Phosphatase (ALP), blood glucose, glycated hemoglobin (HbA1c), triglycerides, total cholesterol, HDL and LDL cholesterol, N-terminal prohormone of brain natriuretic peptide (NT-pro BNP), CRP, albumin and hemoglobin levels were determined.

### Statistical analysis

Quantitative variables are expressed as mean ± standard deviation and qualitative data are expressed as number and percentage. Comparison between analysed patients and excluded patients was performed using Wilcoxon tests, Chi square tests or Fisher exact tests. Differences between before and after bariatric surgery (for clinical and demographic characteristics, dyspnea assessment, 6MWT, arterial blood gases and PFTs) were evaluated using the Student paired t-test and the McNemar Chi-square test as appropriate. Two groups of patients were considered based on the course of the mMRC score after bariatrtic surgery: *i)* the “mMRC improvement group” included patients with a decrease of the mMRC score ≥1, and *ii)* the “no mMRC improvement group” included patients with no change in the mMRC score. Comparisons between patients with mMRC improvement and those without mMRC improvement were performed by univariate analysis, using the Wilcoxon tests and multivariate analysis. For multivariate analysis, colinearity between repiratory variables were studied. A logistic regression will be performed using backward stepwise selection with an exit threshold of 0.10. Age, variation of BMI, variation of CRP, variation of TLC and variation of ERV were included in the multivariate model. Association between improvement of the mMRC score and evolution of lung function tests or laboratory parameters were studied using the Spearman’s correlation coefficient. For all analyses, a p value <0.05 was considered statistically significant. All analyses were performed using SAS version 9.3 (SAS Inc., Cary, NC, USA).

## Results

### Clinical and demographic characteristics

**Fig 1:**
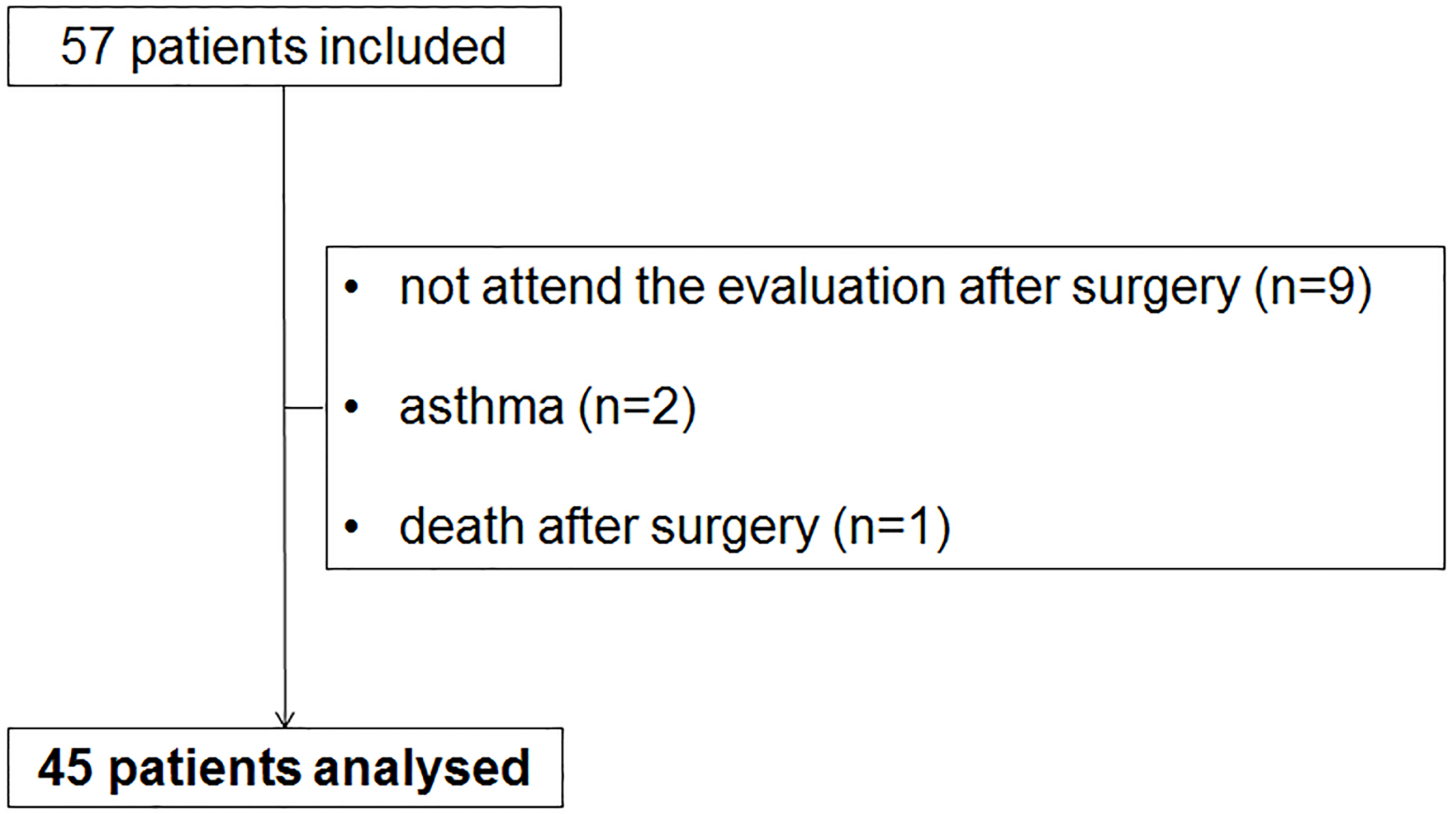
Flowchart of study participants.

Fifty-seven patients were included in the study. Nine patients did not attend the postoperative evaluation, two patients were excluded because of asthma and one patient died just after surgery due to acute respiratory failure. The data for the remaining 45 patients were analyzed (Fig 1).

Clinical and demographic characteristics of the 45 patients analyzed are presented in Table 2. Mean age was 43 ± 11 years. Five men (11%) and forty women (89%) underwent sleeve gastrectomy (31 patients, 69%) or gastric bypass (14 patients, 31%). Mean BMI was 46.2 ± 6.8 kg/m^2^.

**Table 2:** Clinical and demographic characteristics of the 45 patients.

	Patients
**Age (years)**	43 (± 11)
**Sex (M / F)**	5 / 40
**Surgery**	
Sleeve gastrectomy	31 (69%)
Gastric bypass	14 (31%)
**Smoking**	
Current	11 (24%)
Previous	6 (13%)
Never	28 (62%)
Pack-Years	23 (± 16)
**Comorbidities**	
Hypertension	16 (36%)
Diabetes	9 (20%)
Dyslipidemia	13 (29%)
Severe Obstructive Sleep Apnea Syndrome	14 (31%)
**Treatments**	
Antihypertensive drugs	15 (33%)
Oral antidiabetics	8 (18%)
Insulin	3 (7%)
Cholesterol-lowering agents	11 (24%)

Data are expressed as mean (± SD) or number (%)

Abbreviations: M: Male; F: Female

No significant difference was observed between the 12 excluded patients and the 45 analyzed patients in terms of demographic data, weight, BMI, waist measurement, hip measurement, smoking status, comorbidities, dyspnea (according to mMRC and Borg before and after 6MWT), 6MWT, arterial blood gases, PFTs and laboratory parameters.

### Dyspnea assessment before and after bariatric surgery

Results of dyspnea assessment are presented in Table 3. mMRC scores before and after bariatric surgery were available for 42 of the 45 patients of this study. Dyspnea was a very common symptom before surgery, as 90% (n = 38/42 patients) of patients experienced dyspnea in daily living with a mMRC score ≥ 1 and 91% (n = 41/44 patients) of patients experienced dyspnea on exertion with a Borg score ≥ 1 at the end of the 6MWT. Mean mMRC score was 1.5 ± 0.9 before bariatric surgery and was significantly improved to 0.7 ± 0.7 after surgery (p < 0.0001). According to the Borg scale at the end of the 6MWT, dyspnea on exertion was significantly improved by 3.6 ± 2.2 to 2.1 ± 1.9 after surgery (p < 0.0001). Among the patients who experienced dyspnea in daily living according to the mMRC scale (mMRC ≥1) before surgery (n = 38), dyspnea was improved after surgery with 25 patients (66%) exhibiting a decrease in the mMRC score ≥1 (data not shown). Note that only one patient had stopped smoking after surgery.

**Table 3:** Dyspnea assessment before and after bariatric surgery.

Dyspnea assessment	Before surgery	After surgery	p
**mMRC scale (/4) (n = 42)**			
mMRC	1.5 (± 0.9)	0.7 (± 0.7)	**< 0.0001**
mMRC ≥ 1	38 (90%)	25 (59%)	**0.0008**
mMRC ≥ 2	22 (52%)	7 (17%)	**< 0.0001**
**Borg scale (/10) (n = 44)**			
Borg at rest	0.3 (± 0.8)	0.3 (± 1)	0.52
Borg at rest ≥ 1	6 (14%)	4 (9%)	0.32
Borg after 6MWT (/10)	3.6 (± 2.2)	2.1 (± 1.9)	**< 0.0001**
Borg after 6MWT ≥ 1	41 (91%)	30 (67%)	**0.002**
Borg after 6MWT ≥ 5	13 (29%)	7 (16%)	0.08

Data are expressed as mean (± SD) or number (%)

Abbreviations: mMRC: modified Medical Research Council; 6MWT: six-minute walk test.

### Clinical parameters, lung function tests and laboratory parameters before and after bariatric surgery

Results and comparisons of clinical parameters, 6MWT, arterial blood gases, PFTs and laboratory parameters before and after bariatric surgery are presented in Table 4. Bariatric surgery was associated with a mean reduction of BMI from 46.2 ± 6.8 kg/m^2^ to 36.7 ± 5.1 kg/m^2^ (p<0.0001) and a significant improvement of 6MWT parameters (distance covered, SpO2 at rest and at the end of the 6MWT).

**Table 4:** Comparison of clinical parameters, 6-minute walk test, arterial blood gases, pulmonary function tests and laboratory parameters before and after bariatric surgery.

	Before surgery	After surgery		p
**Clinical parameters** (n = 45)				
Weight (kg)	126.7 (± 25)	100.5 (± 18.4)**	*-21%*	**< 0.0001**
Waist measurement (cm)	128 (± 14)	112 (± 15)**	*-20%*	**< 0.0001**
Hip measurement (cm)	142 (± 14)	125 (± 13)**	*-16%*	**< 0.0001**
WM/HM	0.91 (± 0.09)	0.90 (± 0.10)	*-10%*	0.07
Body Mass Index (kg/m²)	46.2 (± 6.8)	36.7 (± 5.1)**	*-20%*	**< 0.0001**
**6-minute walk test** (n = 44)				
6-minute walk test distance (m)	448 (± 63)	467 (± 72)*	*+6%*	**0.01**
SpO2 at rest (%)	97.6 (± 1.7)	98.2 (± 1.3)*	*+0*.*6%*	**0.02**
SpO2 after 6MWT (%)	95.9 (± 2.9)	97.3 (± 2.2)*	*+1*.*4%*	**0.01**
**Arterial blood gases** (n = 42)				
PaO2 (mmHg)	90.1 (± 18.5)	97.9 (± 15.2)*	*+11%*	**0.02**
PaCO2 (mmHg)	37.7 (± 4.2)	36.7 (± 5.1)	*-2%*	0.2
**Pulmonary function tests**				
**Spirometry** (n = 45)				
VC (L)	3.69 (± 0.69)	3.76 (± 0.67)	*+2%*	0.13
VC (% pred)	104 (± 21)	110 (± 15)*	*+4%*	**0.03**
FVC (L)	3.59 (± 0.75)	3.66 (± 0.70)	*+3%*	0.18
FVC (% pred)	104 (± 15)	106 (± 13)	*+3%*	0.16
FEV_1_ (L)	2.85 (± 0.68)	2.90 (± 0.66)	*+3%*	0.15
FEV_1_ (% pred)	97 (± 16)	100 (± 15)*	*+4%*	**0.02**
IC (L)	3.14 (± 0.62)	2.80 (± 0.58)**	*-10%*	**< 0.0001**
IC (% pred)	139 (± 28)	124 (± 28)**	*-10%*	**< 0.0001**
ERV (L)	0.55 (± 0.35)	0.96 (± 0.41)**	*+75%*	**< 0.0001**
ERV (% pred)	48 (± 27)	87 (± 30)**	*+79%*	**< 0.0001**
**Plethysmography** (n = 39)				
RV (L)	1.69 (± 0.58)	1.89 (± 0.56)*	*+27%*	**0.04**
RV (% pred)	103 (± 32)	113 (± 32)	*+24%*	0.06
FRC (L)	2,20 (± 0.48)	2.70 (± 0.62)**	*+23%*	**<0.0001**
FRC (% pred)	82 (± 18)	97 (± 21)**	*+18%*	**<0.0001**
TLC (L)	5.16 (± 0.61)	5.45 (± 0.86)	*+3%*	0.22
TLC (% pred)	103 (± 13)	105 (± 14)	*+4%*	0.18
DLCO (% pred)	100 (± 17)	96 (± 14)*	*-4%*	**0.02**
**Laboratory parameters**				
CRP (mg/L) (n = 36)	12.0 (± 7.5)	6.4 (± 5.7)**	*-45%*	**< 0.0001**
Hemoglobin (g/L) (n = 41)	137 (± 1)	136 (± 1)	*-1%*	0.44
NT-pro BNP (pg/mL) (n = 36)	77 (± 70)	89 (± 84)	*+24%*	0.33

Data are expressed as mean (± SD)

Abbreviations: WM: Waist measurement; HM: Hip measurement; SpO2: Pulse Oxygen Saturation; PaO2: Partial arterial pressure of oxygen; PaCO2: Partial arterial pressure of carbon dioxide; VC: Vital capacity; FVC: Forced vital capacity; FEV_1:_ Forced expiratory volume in one second; IC: Inspiratory capacity; ERV: Expiratory Reserve Volume; RV: Residual Volume; TLC: Total Lung Capacity; DLCO: Carbon Monoxide Diffusing Capacity of the Lung; CRP: C-Reactive Protein; NT-pro BNP: N-terminal prohormone of brain natriuretic peptide.

*p<0.05

**p<0.001

**Fig 2:**
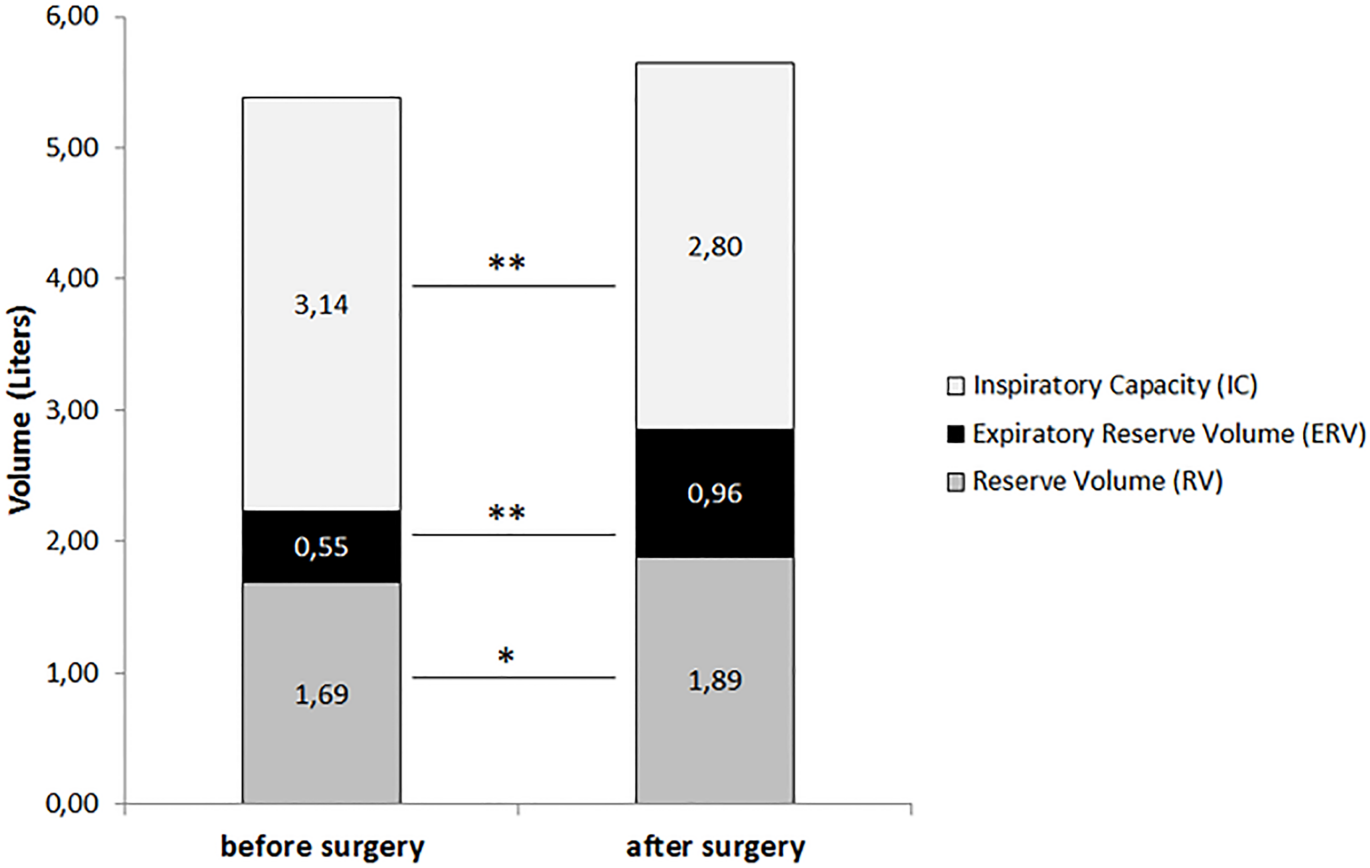
Comparison of Inspiratory Capacity (IC), Expiratory Reserve Volume (ERV) and Reserve Volume (RV) before and after bariatric surgery. *p<0.05; **p<0.0001

Analysis of arterial blood gases showed that PaO2 improved from 90.1 ± 18.5 mmHg to 97.9 ± 15.2 mmHg (p = 0.02). Before surgery, 4 patients (9%) presented with hypoxemia, defined by PaO2 ≤ 70 mmHg, which was corrected in all patients after surgery. Two patients (4%) had obesity hypoventilation syndrome so presented with hypercapnia (PaCO2 ≥ 45 mmHg) before surgery, which persisted after surgery in only one patient (2%) (data not shown). PFTs showed a 75% increase in ERV after surgery (from 0.55 ± 0.35 L to 0.96 ± 0.41 L) (p < 0.0001), a 27% increase in RV (from 1.69 ± 0.58 L to 1.89 ± 0.56 L) (p = 0.04) and a 10% decrease in IC (from 3.14 ± 0.62 L to 2.80 ± 0.58 L) (p < 0.001) (Fig 2).

Serum CRP decreased significantly after bariatric surgery (12.0 ± 7.5 mg/L to 6.4 ± 5.7 mg/L, p < 0.0001). Serum HbA1c (5.7 ± 0.9% to 5.4 ± 1.2%, p < 0.001) and GammaGT (30 ± 17 IU/L to 18 ± 10 IU/L, p < 0.0001) were significantly lower after bariatric surgery. Serum albumin (40.7 ± 3 g/L to 42.7 ± 2.3 g/L, p < 0.0001) and HDL cholesterol (1.1 ± 0.3 mmol/L to 1.2 ± 0.3 mmol/L, p < 0.0001) were significantly increased after surgery (data not shown).

### Relationships between dyspnea improvement, respiratory and laboratory parameters

Thirty-eight patients (90%) experienced dyspnea in daily living before bariatric surgery (mMRC ≥ 1). After bariatric surgery, dyspnea, assessed by according the mMRC scale, was improved in 25/38 patients (66%) (“mMRC improvement group”) and was not improved in 13/38 patients (34%) (“no mMRC improvement group”). No patient experienced more severe dyspnea after surgery according to the mMRC scale.

No significant difference was observed between these two groups of patients (“mMRC improvement group” and “no mMRC improvement group”) at baseline before surgery (data not shown).

Variations of clinical, respiratory and laboratory parameters before and after bariatric surgery were compared between these 2 groups of patients (“mMRC improvement group” versus “no mMRC improvement group”) (Table 5). The most interesting results in terms of PFTs were variations in ERV, IC and TLC. ERV was more markedly improved in the group of patients in whom dyspnea was improved after surgery than in the group of patients with no improvement of dyspnea (respectively +0.49 ± 0.3 L and +0.17 ± 0.32 L; p = 0.01). Inversely, a more marked decrease in IC was observed in the “mMRC improvement group” than in the “no mMRC improvement group” (-0.44 ± 0.40 and -0.02 ± 0.41 L; p = 0.01). TLC was slightly decreased in the “mMRC improvement group” and increased in the “no mMRC improvement group” (-0.12 ± 0.77 L and +0.48 ± 0.46 L (p = 0.01)).

**Table 5:** Comparison of patients without mMRC improvement and with mMRC improvement after bariatric surgery based on variations of clinical, respiratory and laboratory parameters in dyspneic patients before surgery according mMRC scale (n = 38).

	No mMRC improvement (n = 13)	mMRC improvement (n = 25)	p	
**Clinical parameters**				
Δ weight (kg)	-26.1 (± 10.1)	-25.7 (± 13.6)	0.78	
Δ WM (cm)	-17 (± 11)	-16 (± 10)	0.82	
Δ HM (cm)	-14 (± 13)	-14 (± 9)	0.88	
Δ WM/HM	-0.03 (± 0.09)	-0.02 (± 0.08)	0.61	
Δ BMI (kg/m²)	-9.4 (± 3.5)	-9.3 (± 4.4)	0.90	
**6-minute walk test**				
Δ 6MWT distance (m)	33 (± 55)	19 (± 53)	0.34	
Δ SpO2 at rest (%)	0.7 (± 2.2)	0.7 (± 1.4)	0.45	
Δ SpO2 after 6MWT (%)	2.1 (± 3.6)	0.7 (± 3.2)	0.15	
**Arterial blood gases**				
Δ PO2 (mmHg)	12.2 (± 12.4)	5.3 (± 21.9)	0.57	
Δ PCO2 (mmHg)	-1.8 (± 5.0)	-0.7 (± 5.4)	0.97	
**Pulmonary function tests**				
Δ VC (L)	0.15 (± 0.42)	0.06 (± 0.20)	0.68	
Δ VC (% pred)	4 (± 13)	7 (± 18)	0.60	
Δ FVC (L)	0.12 (± 0.41)	0.08 (± 0.30)	0.98	
Δ FVC (% pred)	3 (± 13)	2 (± 8)	0.89	
Δ FEV1 (L)	0.07 (± 0.32)	0.07 (± 0.20)	0.86	
Δ FEV1 (% pred)	3 (± 11)	4 (± 7)	0.99	
Δ IC (L)	-0.02 (± 0.41)	-0.44 (± 0.40)*	**0.01**	
Δ IC (% pred)	-1 (± 19)	-19 (± 17)*	**0.01**	
Δ ERV (L)	0.17 (± 0.32)	0.49 (± 0.35)*	**0.01**	
Δ ERV (% pred)	17 (± 32)	45 (± 31)*	**0.01**	
Δ RV (L)	0.44 (± 0.36)	0.01 (± 0.68)	0.08	
Δ RV (% pred)	27 (± 26)	-1 (± 38)	0.06	
Δ TLC (L)	0.48 (± 0.46)	-0.12 (± 0.77)*	**0.01**	
Δ TLC (% pred)	10 (± 9)	-2 (± 15)*	**0.02**	
Δ DLCO (% pred)	1 (± 13)	-7 (± 13)	0.07	
**Laboratory parameters**				
Δ CRP (mg/L)	-2.1 (± 3.2)	-7.2 (± 5.9)*	**0.01**	
Δ Haemoglobin (g/L)	1 (± 12)	- 2 (± 8)	0.67	
Δ NT-pro BNP (pg/mL)	11.8 (± 117.5)	6.9 (± 41.2)	0.72	

Data are expressed as mean ± SD.

Abbreviations: WM: Waist measurement; HM: Hip measurement; BMI: Body Mass Index; 6MWT: six-minute walk test; SpO2: Pulse Oxygen Saturation; PaO2: Partial arterial pressure of oxygen; PaCO2: Partial arterial pressure of carbon dioxide; VC: Vital capacity; FVC: Forced vital capacity; FEV_1:_ Forced expiratory volume in one second; IC: Inspiratory capacity; ERV: Expiratory Reserve Volume; RV: Residual Volume; TLC: Total Lung Capacity; DLCO: Carbon Monoxide Diffusing Capacity of the Lung; CRP: C-reactive protein; NT-pro BNP: N-terminal prohormone of brain natriuretic peptide.

*p<0.05

In terms of laboratory parameters, CRP was significantly more decreased in the “mMRC improvement group” compared to the “no mMRC improvement group” (-7.2 ± 5.9 mg/L versus -2.1 ± 3.2 mg/L; p = 0.01).

Among the 38 patients who experienced dyspnea according to the mMRC scale before bariatric surgery, we found a correlation between improvement of the mMRC score and increased ERV (L) (r = -0.37, p = 0.02), as well as between improvement of the mMRC score and decreased IC (L) (r = +0.34, p = 0.04) and between improvement of the mMRC score and change in TLC (r = + 0.40, p = 0.02). A correlation was also observed between improvement of the mMRC score and decreased CRP (r = +0.55, p = 0.001). The multivariate analysis including age, variation of BMI, variation of CRP, variation of TLC and variation of ERV in the model, showed that only the increase in ERV was associated with improvement of the mMRC score (p = 0.04).

## Discussion

This is the first study to specifically assess the relationships between dyspnea in daily living according to the mMRC scale and lung function tests and laboratory parameters, before and after bariatric surgery in patients with obesity. As expected, our study shows that weight loss associated with bariatric surgery is associated with an improvement of dyspnea in daily living and an increasing of ERV. In particular, our study highlights an association between dyspnea improvement according to the mMRC scale and increased ERV after bariatric surgery in patients with obesity who experienced dyspnea before surgery.

In this study, 90% of patients experienced dyspnea in daily living according to the mMRC scale before bariatric surgery, confirming the high prevalence of dyspnea already observed in other studies in patients with obesity [6,7]. Data supporting improvement of dyspnea in daily living after bariatric surgery are scarce. In a large cohort of patients with obesity, bariatric surgery was shown to significantly improve self-reported dyspnea during activities of daily living (i.e. climbing two flights of stairs) [20]. The present study demonstrates that bariatric surgery improves dyspnea in daily living with improvement of dyspnea according to the mMRC scale in 66% of patients who experienced dyspnea before surgery. This result is supported by a significant improvement in dyspnea on exertion according to the Borg scale at the end of the 6MWT after bariatric surgery in our study (Table 3).

The 6MWT is an objective and easy-to-use measure of functional capacity, which is extensively used in patients with heart or lung disease [21]. More recently, the 6MWT has been performed in patients with obesity undergoing bariatric surgery. Our study found a significant improvement in the distance covered on the 6MWT, but less than that reported in other studies, in which a mean increase of 70 to 80 m was observed [12,13]. This discordant result is probably related to the more marked weight loss observed after bariatric surgery in these studies.

Mild hypoxemia is reported and has been associated with abdominal obesity, which is more common in morbidly obese men [22]. However, weight loss has a variable effect on blood gases, as some studies have reported improvement of PaO2, while others have reported no change [11,23]. In our study population, mainly composed of women, mean PaO2 was normal and was significantly improved after bariatric surgery (Table 4), which may be due to a combination of ventilation-perfusion mismatch and hypoventilation. The main cause of ventilation-perfusion mismatch in obesity is underventilation of well-perfused lower lung regions [24]. The fact that PaCO2 did not change after weight loss in our study (Table 4) suggests that ventilation-perfusion mismatch was probably more severe than hypoventilation.

Our study also highlights a slight decrease in DLCO after bariatric surgery (Table 4). DLCO is usually preserved in obesity [25], but some studies have described a slight increase in DLCO in patients with obesity (3,26)[3,26]. Saydain *et al* [26] explained that DLCO in obesity is higher because of an increase in intrathoracic blood volume, which could explain why DLCO decreased slightly with weight loss in our study (Table 4).

As expected, the most consistently reported modification in lung volume before bariatric surgery consists of decreased ERV (Table 4). There is strong evidence to suggest that significant weight loss improves ERV in both mild-to-moderate and severe obesity [11,27–29]. As expected, in our study, we also found a marked improvement in ERV after weight loss associated with bariatric surgery (+75%). Note that, as previously described [11,30], RV was also moderately increased (+27%) after bariatric surgery, IC was slightly decreased (-10%) and TLC was not significantly increased.

Very interestingly, dyspneic patients in whom dyspnea was improved after bariatric surgery had a more marked increase in ERV and a more marked decrease in IC and a decrease in TLC compared to patients in whom dyspnea was not improved, suggesting that the predominant mechanism of dyspnea in obesity related to lung volumes is not the decrease in TLC (which is also usually normal in obesity), but the decrease in ERV (and consequently the increase in IC). It is noteworthy that patients in whom dyspnea was improved after bariatric surgery (“mMRC improvement group”) presented a more marked increase in ERV, but similar weight loss to that of patients with no improvement of dyspnea (“no mMRC improvement group”). Although the decrease in waist and hip circumferences was similar in the “mMRC improvement group” and “no mMRC improvement group”, it is possible that fat distribution was different between the two groups. Body composition and fat distribution affect lung function, especially when excess fat is located in the chest and abdomen [31,32]. Fat deposition on the hips would be unlikely to have any direct mechanical effect on the lungs or chest wall. Abdominal fat displaces the diaphragm into the abdomen and impedes its downward movement during inspiration [33]. Fat deposition on the chest wall may impede expansion and excursion of the rib cage during inspiration, either via a direct loading effect or by changing intercostal muscle function. Several studies have used dual-energy X-ray absorptiometry (DXA) to quantify fat and lean mass in different regions of the body and have reported negative correlations between upper body fat distribution and ERV [32]. Waist and hip measurements are easy to use in clinical practice to evaluate central obesity, but provide less information than DXA, which is why it would be interesting to evaluate the relationships between improvements of dyspnea and ERV after weight loss and fat distribution assessed by DXA.

One of the strengths of this study is the assessment of the relationships between mMRC improvement after bariatric surgery and PFTs, arterial blood gases, 6MWT and laboratory parameters. To our knowledge, this is the first study to report an association between dyspnea improvement/increased ERV, which may contribute to our understanding of the mechanisms of dyspnea in obese subjects. Nevertheless, improvement of dyspnea after bariatric surgery is probably multifactorial and factors other than increased ERV are also likely to contribute to this symptom, reflecting the complex mechanisms of dyspnea. It would be interesting to assess the relationships between dyspnea and respiratory muscle performance (PImax and SNIP), which may be decreased in subjects with obesity [8] and the relationships between dyspnea and respiratory drive parameters [34]. Furthermore, dyspnea on exertion also appears to be associated with a more marked increase in the oxygen cost of breathing during exercise [35], but this parameter was not assessed in this study. Another limitation of our study is that we did not take psychiatric parameters into account, for example anxiety or depression, which could increase dyspnea sensations [36]. Finally, this study was conducted in a single center in a relatively small number of patients. Given the low number of patients and the low number of men in this study, a comparative analysis by gender was not possible. Our results may then not be applicable to men. There is also a potential selection bias of patients because only patients attending the postoperative evaluation were considered in this study. We can not exclude that some patients with no mMRC improvement after bariatric surgery may have been removed from this analysis. For all these reasons, the results of this study should be interpreted cautiously. Further larger multicentric studies are needed to confirm these results.

## Conclusion

This prospective study shows that bariatric surgery improves dyspnea in daily living according to the mMRC scale in patients with obesity. It highlights an association between improvement of dyspnea after bariatric surgery and increased ERV, suggesting that improvement of dyspnea after weight loss associated with bariatric surgery could be partly related to increased Expiratory Reserve Volume which is the lung volume abnormality most frequently associated with obesity.

## Supporting information

S1 File Supporting information: Database. (XLSX)
